# Total saponins from quinoa bran alleviate high‐fat diet‐induced obesity and systemic inflammation via regulation of gut microbiota in rats

**DOI:** 10.1002/fsn3.2984

**Published:** 2022-07-22

**Authors:** Wei Li, Yu Song, Ya‐Nan Cao, Le‐Le Zhang, Gang Zhao, Ding‐Tao Wu, Liang Zou

**Affiliations:** ^1^ School of Preclinical Medicine Chengdu University Chengdu China; ^2^ Key Laboratory of Coarse Cereal Processing, Ministry of Agriculture and Rural Affairs, Sichuan Engineering & Technology Research Center of Coarse Cereal Industralization, School of Food and Biological Engineering Chengdu University Chengdu China

**Keywords:** inflammation, insulin resistance, intestinal flora, metabolic endotoxemia, quinoa saponins

## Abstract

In recent years, biologically active ingredients derived from natural plants or functional foods have raised considerable interests for its anti‐obesity effect. Quinoa (*Chenopodium quinoa* Willd.) is a traditional staple food in the Andean regions of Peru which contains a variety of bioactive components. This study aimed to investigate the potential therapeutic effect of total saponins extracted from quinoa bran (TSQ) on obese rats and explore whether the underlying mechanism is related to intestinal microbiota. Results showed that TSQ could decrease the body weight gain and visceral fat accumulation in the obese rats. Moreover, trends in ameliorating insulin resistance and improved glucose tolerance were observed. Indeed, Pearson's correlations analysis revealed that the variation in gut microbial composition was highly correlated to insulin resistance, IL‐6, and LPS levels. Collectively, these results suggest that the prevention of obesity and inflammation by TSQ may be mediated by the modulation of gut microbial composition.

## Graphical abstract

TSQ exhibited the most promising effects on suppressing body weight gain and visceral white adipose accumulation, improving glucose intolerance and insulin resistance, and attenuating metabolic endotoxemia, systemic inflammation, and the serum lipid profile. TSQ modulated gut microbiota dysbiosis by increasing the expansion of beneficial bacteria and repressing the abundance of harmful bacteria. The changes in gut microbiota induced by TSQ are correlated with obesity‐related metabolic disorders (insulin resistance, LPS, and inflammation levels), suggesting that the prevention of obesity and inflammation by TSQ may be mediated by component modulation of the gut microbiota.

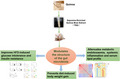

## 1 INTRODUCTION

Obesity is a serious and increasing health problem, which is considered to be strongly associated with chronic metabolic diseases, including type 2 diabetes mellitus (T2DM), hyperlipidemia, hypertension, and nonalcoholic steatohepatitis (Han & Lean, 2016). The fundamental feature of obesity is the extreme disequilibrium between energy uptake and expenditure, leading to abnormal excessive growth of adipose tissue (Lin & Lin‐Shiau, 2006). Growing evidence suggests that the typical characteristic of obesity is low‐grade chronic inflammation, which may trigger a cascade of detrimental health consequences such as insulin resistance, T2DM, and dyslipidemia (Masoodi et al., 2015).

Gut microbiota has been documented to play a critical role in systemic metabolism by modulating energy harvest, fat storage, and immune response of its host (Nicholson et al., 2012; Shen et al., 2013). Recently, it has been increasingly recognized that gut microbiota plays an important role in the pathogenesis of obesity and metabolic diseases (Cani et al., 2008). In normal individuals, gut microbiota acts as an important organ of host by digesting food components and regulating energy homeostasis. However, the balance structure of gut microbiota will be altered by high‐fat diet (HFD) consumption and trigger a series of physiological problems, including chronic low‐grade inflammation, gut barrier dysfunctions, metabolic endotoxemia, insulin resistance, and obesity (Brugman et al., 2006). It has been confirmed that regulating the structure of intestinal microbiota by dietary or drug intervention had a beneficial effect on alleviation of obesity and its associated inflammation both in humans and animals. For example, previous research indicated that the improved abundance of *Bifidobacterium* by oral administration of insulin‐type fructans significantly prevented obesity in HFD‐induced mice (Cani et al., 2007). Therefore, gut microbiota is a potential target for medication or dietary interventions in obese individuals (Jia et al., 2008; Zhao & Shen, 2010). In recent years, intensive researchers have demonstrated that the secondary metabolites of plants can be used to restore the disordered gut microbiome both in humans and animals, such as flavonoids, polyphenols, and polysaccharides (Hu et al., 2017; Wu, Feng, et al., 2022; Wu, He, et al., 2022). Collins et al. (2016) found that polyphenol extracts of grapes could modulate the gut bacterial community structure, decrease markers of inflammation, and ultimately reduce body weight gain and fat accumulation in obese mice. Like polyphenols, saponins are a diverse group of biologically active compounds derived from medicinal plants or edible plants. Herbal saponins exhibit a wide range of physiological and pharmacological activities, such as antitumor, immunomodulation, hepatoprotective, cardiovascular protection, cholesterol‐lowering, anti‐inflammatory, and antiviral activities (Francis et al., 2002; Sparg et al., 2004). Owing to their extensive activities, saponins have been used as natural products for the prevention and treatment of obesity. Multiple studies have indicated that saponins from *Panax ginseng*, *Panax japonicus*, and *Platycodi radix* are the most potent and well‐recognized natural anti‐obesity active compounds in animal models (Attele et al., 2002; Yun, 2010).

Quinoa has been widely regarded as the extremely nutritious corn around the world due to both numerous macronutrients and micronutrients of its grain, such as high‐quality protein, fatty acids, polysaccharides, vitamins, and minerals (Vega‐Gálvez et al., 2010). Despite its healthy nutritional composition, phytochemicals are considered to be the major bioactive constituents in quinoa, such as saponins, flavonoids, phenolic acids, betalains, and carotenoids. For instance, an experimental study has revealed that the antioxidant capacity of quinoa is related to its high content of phenolics (Abderrahim et al., 2015). Meanwhile, various researches have proved the antioxidant, anti‐inflammatory, and other health‐promoting effects of quinoa in vivo and in vitro (Tang & Tsao, 2017). Currently, triterpene saponins have attracted wide attention, which have been found in various parts of quinoa, especially in the hull (Kuljanabhagavad et al., 2008). The quinoa saponin is usually removed by dehulling or soaking before eating because of bitter‐tasting and antinutritional properties (Ma et al., 1989; Ruales & Nair, 1993). However, the health benefits of quinoa saponins have attracted more attention and interest, such as anti‐inflammatory (Yao, Shi, & Ren, 2014), antifungal (Stuardo & San Martín, 2008), anticancer (Hu et al., 2017), immune‐regulatory, and antioxidant activities (Yao, Shi, & Ren, 2014).

Recently, our research group found that saponins extracted from quinoa bran possessed anti‐inflammatory activity, which was closely related to the inhibition of the release of inflammatory cytokines in vitro (Yao, Shi, & Ren, 2014). However, the anti‐inflammatory and anti‐obesity activities of total saponins extracted from quinoa bran (TSQ) in vivo and the potential mechanisms of action are still not clear. Therefore, the purpose of this study is to explore the effect of TSQ on diet‐induced obesity in rats and to determine whether the resultant prevention of obesity and anti‐inflammatory effects are mediated by modulating the gut microbial composition.

## 2 MATERIALS AND METHODS

### 2.1 Chemicals and reagents

*Chenopodium quinoa* bran was kindly donated by Professor Guixin Ren (Beijing, China), the president of quinoa Association of China. Serum biochemical parameters were determined by using assay kits. These assay kits were obtained from BioSino Bio‐technology and Science, Inc. (Beijing, China). ELISA kits were obtained from ExCell Biology, Inc. (Shanghai, China). Blood glucose was assessed by a standard glucometer (Accu‐chek® Performa, Roche Diagnostics, Germany). All other chemicals and reagents are analytical grade.

### 2.2 Animals

Male Sprague Dawley (SD) rats (180 ± 20 g) were obtained from Da Shuo Biological Technology Co., Ltd. (Chengdu, China). The rats were housed in standard polypropylene cages (four rats per cage) and maintained under controlled room temperature (24 ± 2°C) and humidity (55 ± 5%) with a 12/12‐h light/dark cycle.

### 2.3 Preparation of total saponins from quinoa bran

Aqueous two‐phase extraction system (ATPS) was applied for the extraction of total saponins from quinoa bran. The ATPS of ethanol/K_2_HPO_4_ was prepared as follows: 12.5 g of K_2_HPO_4_ was dissolved in 250 ml of 75% v/v aqueous ethanol solution and then mixed well by a vortex stirrer to spontaneously separate into two phases. Then TSQ was prepared according to a modified method as previously reported (Yao, Yang, et al., 2014). First, the air‐dried quinoa bran was finely ground into powder. Quinoa bran powder (30.0 g) was placed in a Soxhlet apparatus and extracted at 80°C for 2 h with petroleum ether to remove pigments and lipids, then dried as ample for the following extraction. Immediately afterward, the sample powder was put into a beaker, added 750 ml of the ATPS, and then extracted by ultrasonication for 1 h (KQ‐800, Kunshan Ultrasonic Instruments Co., Ltd., Kunshan, China). The extraction procedure was repeated twice, and the filtrates were combined. Furthermore, the filtrate was concentrated and extracted with *n*‐butanol saturated with water. The extract was concentrated and dried by vacuum from the *n*‐butyl alcohol solution, and further purified with D101 macroporous absorptive resin and lyophilized to obtain TSQ extract powder.

### 2.4 Determination of total saponins

The content of total saponins in TSQ was determined by UV–visible colorimetry with oleanolic acid as a standard substance (Weng et al., 2014). In brief, 0.5 ml of TSQ solution was added into a centrifuge tube and then the solvents were evaporated at 60°C in a water bath. Then, 0.2 ml of 5% vanillin–glacial acetic acid solution and 0.8 ml of perchloric acid were added into the residue. Thereafter, 2.5 ml of glacial acetic acid was added after incubating in a 60°C water bath for 15 min and quickly cooled in ice water. The absorbance of the mixture was measured by spectrophotometry at a maximum absorption wavelength of 554 nm. The total saponin content was expressed as a percentage.

### 2.5 Hemocompatibility test

The hemolytic effect of TSQ was evaluated according to the method described by Yang et al. (2019). Briefly, 0.5 ml of TSQ was mixed and shaken gently with 2% of red blood cells (RBCs; 0.5 ml) and then incubated in a water bath at 37°C for 3 h. Subsequently, the incubated mixture was centrifuged at 1500 rpm for 5 min at 4°C. Next, 200 μl solution was removed and placed in 96‐well plates. The absorbance was detected at 540 nm with a microplate reader (Synergy H1; Bio‐Tek, USA). Negative control (−) and positive control (+) were prepared by PBS and deionized water, respectively. Three parallels were set for each sample. Percent hemolysis was calculated using the following equation: Percent hemolysis = *A*_s_−*A*_n_/*A*_p_−*A*_n_ × 100%, where *A*_S_ is the absorption value of the sample, *A*_n_ is the absorption value of the negative control, and *A*_p_ is the absorption value of the positive control.

### 2.6 Acute toxicity assay

The acute toxicity assay of TSQ was performed according to OECD guideline no. 425: Oral Acute Toxicity – Up‐and‐Down Procedure (OECD, 2008). Five male rats per treatment group were used in this assay, being: (1) negative control group, treated with oral saline, and (2) test group, treated with oral TSQ. The extract was administered by gavage at a dose of 2000 mg/kg body weight to a single rat. Sequentially, at 48 h intervals, the same dose was administered to four more rats. In parallel, the group of five male rats from the vehicle control was treated with saline to compare with the test group. The rats were observed for 2 weeks for any signs of morbidity or mortality.

### 2.7 Animal studies

All animal procedures were performed according to the Guidelines for the Care and Use of Experimental Animals and were approved by the Experimental Animals Ethics Committee of the Chengdu University. After 7 days of acclimatization, 8 SD male rats were fed with normal control diets (NCD) as a control from the beginning. Then, all rats, except those of the normal control group, were fed with HFD (consisting of 24% total fat, 24% protein, and 48% carbohydrates, 45% calories from fat) to the end of the experiment (total 11 weeks). After 4 weeks of HFD feeding, the rat's serum TC, TG, LDL‐C, and HDL‐C levels and body weight were measured to assess the establishment of diet‐induced obesity rats. There was a statistically significant increase in body weight (*p* < .05) and serum levels of triacylglycerol (TG) (*p* < .05), low‐density lipoprotein cholesterol (LDL‐C) (*p* < .05), and high‐density lipoprotein cholesterol (HDL‐C) in the HFD group compared to the NCD group. The experimental results suggest that the diet‐induced obesity rat model was successfully prepared, which can be utilized for further experiments. Sixteen male HFD rats were randomly divided into two groups and started to receive TSQ (100 mg/kg) or 0.5% of carboxymethylcellulose sodium (CMC‐Na) by gavage for an additional 7 weeks. All rats were maintained on their previous diets during the experiment. The rats had free access to water and food during this period. Body weight was recorded every week. At the end of the experiment, fresh fecal samples were collected and immediately frozen at −80°C for future analyses. Hereafter, oral glucose tolerance test (OGTT) was performed and then these rats were anesthetized with chloral hydrate (10 mg/kg) prior to the overnight fasting. Blood samples were collected by femoral arteries and centrifuged at 3000 rpm for 15 min at 4°C, and finally the plasma was separated and stored at −80°C until analysis. Liver, epididymal, and retroperitoneal white adipose tissues (WAT) were harvested, frozen immediately in liquid nitrogen, and stored at −80°C.

### 2.8 Oral glucose tolerance test

Oral glucose tolerance test was performed before the start and after 11 weeks of feeding and gavage. Twelve hours fasted rats were given an oral glucose load (2 g/kg of body weight) and blood samples were collected from the tail vein. The blood glucose was assessed by a standard glucometer (Accu‐chek® Performa, Roche Diagnostics, Germany) at 0, 30, 60, 90, and 120 min after oral glucose load. The total glucose area under the curve (AUC) between 0 and 120 min represented the magnitude of the glucose response.

### 2.9 Serum biochemical measurements

The levels of TG, total cholesterol (TC), HDL‐C, and LDL‐C in serum samples were assayed according to the manufacturer's instructions (BioSino Bio‐technology and Science, Inc., Beijing, China). The levels of fasting insulin, lipopolysaccharide (LPS), interleukin 1β (IL‐1β), interleukin 6 (IL‐6), and TNF‐α were measured using ELISA kits (ExCell Biology, Inc., Shanghai, China). The homeostasis model assessment of insulin resistance (HOMA‐IR) was calculated by the following formula: fasting insulin (mU/L) × fasting glucose (mmol/L)/22.5.

### 2.10 Fecal DNA extraction and sequencing

DNA was extracted from fecal samples using the ZR Fecal DNA MiniPrep Kit (Zymo Research Corporation, Irvine, CA, USA) according to the manufacturer's instructions. The V_3_–V_4_ hypervariable region of the 16S rDNA gene was amplified using the extracted bacterial genomic DNA (10 ng) template and primers 341F‐806R. The PCR analysis was conducted on a thermocycler PCR system (ABI GeneAmp PCR System 9700, Applied Biosystems, CA, USA) using 20 μl of TransStart FastPfu DNA Polymerase. High‐throughput pyrosequencing of the PCR products from different samples were performed on a Miseq Illumina Sequencing Platform at TGS Co., Ltd. (Shenzhen, China). All high‐quality reads were clustered into operational taxonomic units (OTU) using USEARCH (v7.0.1090) based on a 97% similarity. For alpha diversity analysis, we rarified the OTU to several metrics, including OTU rank curves, Shannon index, Chao1 index, observed species index, and PD whole tree index. For beta‐diversity analysis, heatmap of RDA‐identified key OTUs and partial least squares discrimination analysis (PLS‐DA) were performed using QIIME.

### 2.11 Statistical analysis

All statistical analyses were performed using SPSS 22.0 statistical software (IBM Corp, Armonk, NY, USA). The relative abundance of gut microbiota in each sample and other obtained data were given as the mean ± SD. The results were analyzed by one‐way analysis of variance (ANOVA). Correlations between parameters were assessed by Pearson's correlation test. ^▲/^*p < .05, ^▲▲/^**p < .01, and ^▲▲▲/^***p < .001 were considered statistically significant.

## 3 RESULTS

### 3.1 Extraction of TSQ and its content of total saponins

Ultrasonic‐assisted ATPS was applied for the extraction of total saponins from quinoa bran. The dry powder was extracted by using ATPS to obtain TSQ and the extraction yield was 9.80%. The content of total saponins of TSQ extracts were calculated by standard curve obtained with vanillin–perchloric acid colorimetric method. After the purification by D101 macroporous absorptive resin chromatography, the purity of TSQ was raised to 84.70% according to the linear relationship *Y* = 20.139*X* − 0.0265 of the standard (oleanolic acid).

### 3.2 Saponin‐related toxicity tests

In this study, according to the guide of OECD, the acute toxicity test method was used to evaluate the safety of TSQ in vivo. Up‐and‐down procedure was applied to assess the acute oral toxicity of TSQ. The results showed that administration of the vehicle or TSQ (2000 mg/kg) in male rats did not produce any signs of intoxication, and all animals survived through the study period of 14 days.

**FIGURE 1:**
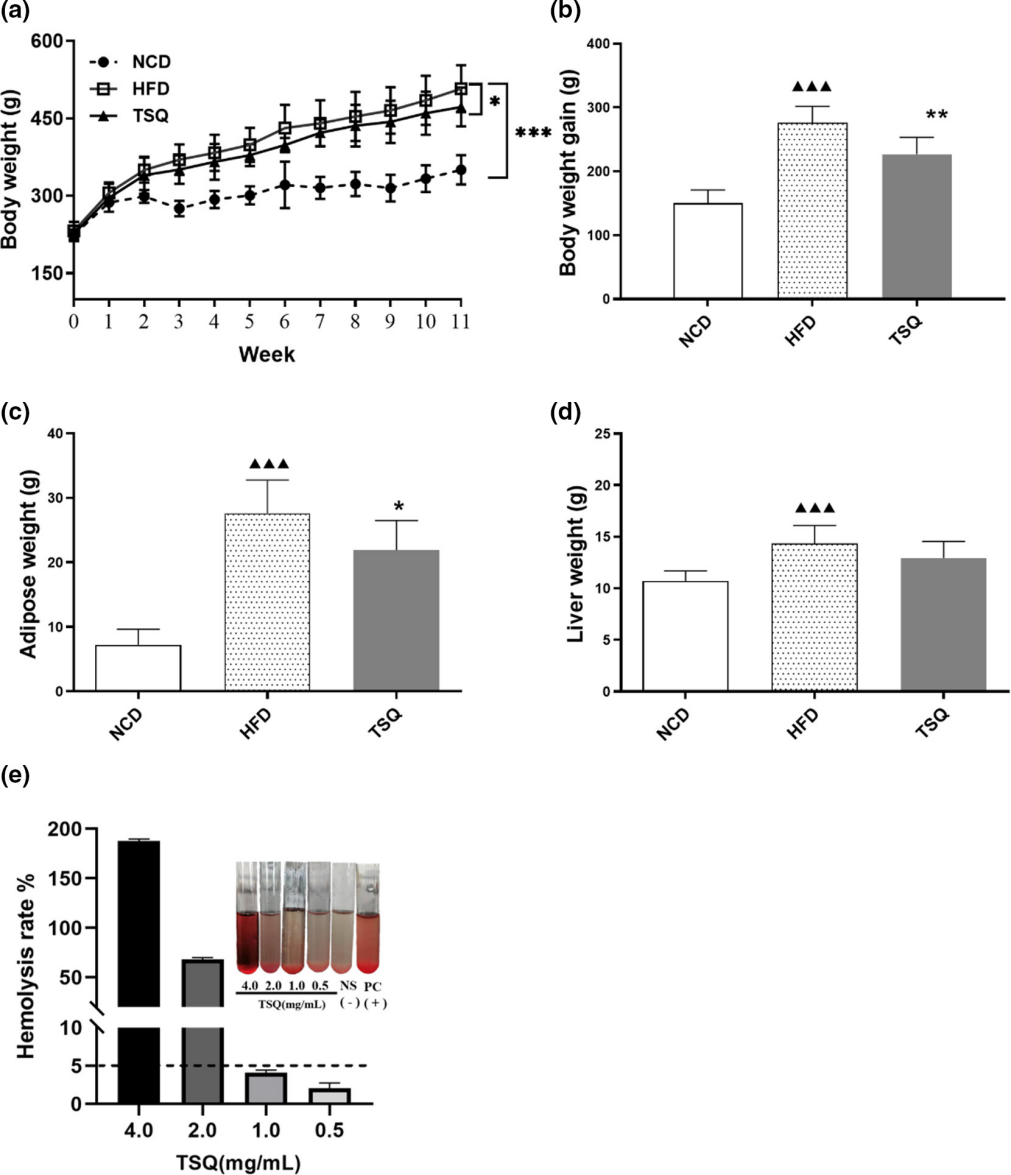
TSQ prevents body weight gain and adipose tissue accumulation in diet‐ induced obese rats and in vitro hemolysis. (a) Growth curves, (b) body weight gain, (c) weight of adipose tissue, and (d) weight of liver of rats in normal diet (NCD), high‐fat diet (HFD), and HFD supplemented with TSQ groups. (e) Hemolysis rate. ^▲▲▲^ *p* < .001, statistically significant differences with NCD group; **p < .01, *p < .05, statistically significant differences compared with HFD group

As is well‐known, saponins are susceptible to combine with cholesterol and generate insoluble complexes in blood. As a consequence, these complexes are tended to destroy the permeability of erythrocytes, which produce the cardinal features of intravascular hemolysis and anemia observed. Therefore, we evaluated the hemolysis of TSQ in vitro. As shown in Figure 1f, the positive control group (Tube PC) was red and clear without any cell residue at bottom, it suggested that erythrocytes were totally hemolytic. With the decrease in concentration of TSQ, the red blood cells sank and the supernatants were colorless and clear compared with the negative control group (Tube NS). Further calculations revealed that the hemolysis rate of TSQ at the concentration less than 1.0 mg/ml was lower than 5% and the structure of erythrocytes was little changed.

### 3.3 TSQ prevents diet‐induced body weight gain and visceral white adipose accumulation

Administration of TSQ significantly reduced HFD‐induced body weight gain and visceral white adipose accumulation, resulting in a lower final body weight than HFD group. Before the initiation of the experiment, the body weights among the three groups were not significantly different. After a high‐fat diet, an obvious body weight increase was observed in the HFD groups compared with the NCD group over time. Body weight gain in the TSQ group was significantly lower than the HFD group after 11 weeks intervention (Figure 1a). At the end of the experiment, rats in the HFD group gained more body weight than rats in the NCD group (275.83 ± 25.87 g vs. 150.10 ± 20.88 g*, p* < .001) (Figure 1b). However, the body weight gain of rats in TSQ group was significantly lower than that in HFD group (226.97 ± 26.18 g vs. 275.83 ± 25.87 g*, p* < .01) (Figure 1b). High‐fat diet caused more rapidly accumulation of adipose tissue in HFD group than NCD group (27.62 ± 5.14 g vs. 7.20 ± 2.45 g*, p* < .001) (Figure 1c). However, TSQ supplementation significantly reduced the rate of fat accumulation compared with that of the HFD group (21.91 ± 4.58 g vs. 27.62 ± 5.14 g*, p* < .05) (Figure 1c). The final body adipose tissue weight in the HFD group was almost four times higher than of the NCD group (Figure 1c), while TSQ treatment significantly reduced adipose accumulation. In terms of liver weight, values were significantly higher in the HFD group than that of the NCD group. However, the continued consumption of TSQ could reduce the liver weight with no significant differences (Figure 1d).

### 3.4 TSQ improves HFD‐induced glucose intolerance and insulin resistance

**FIGURE 2:**
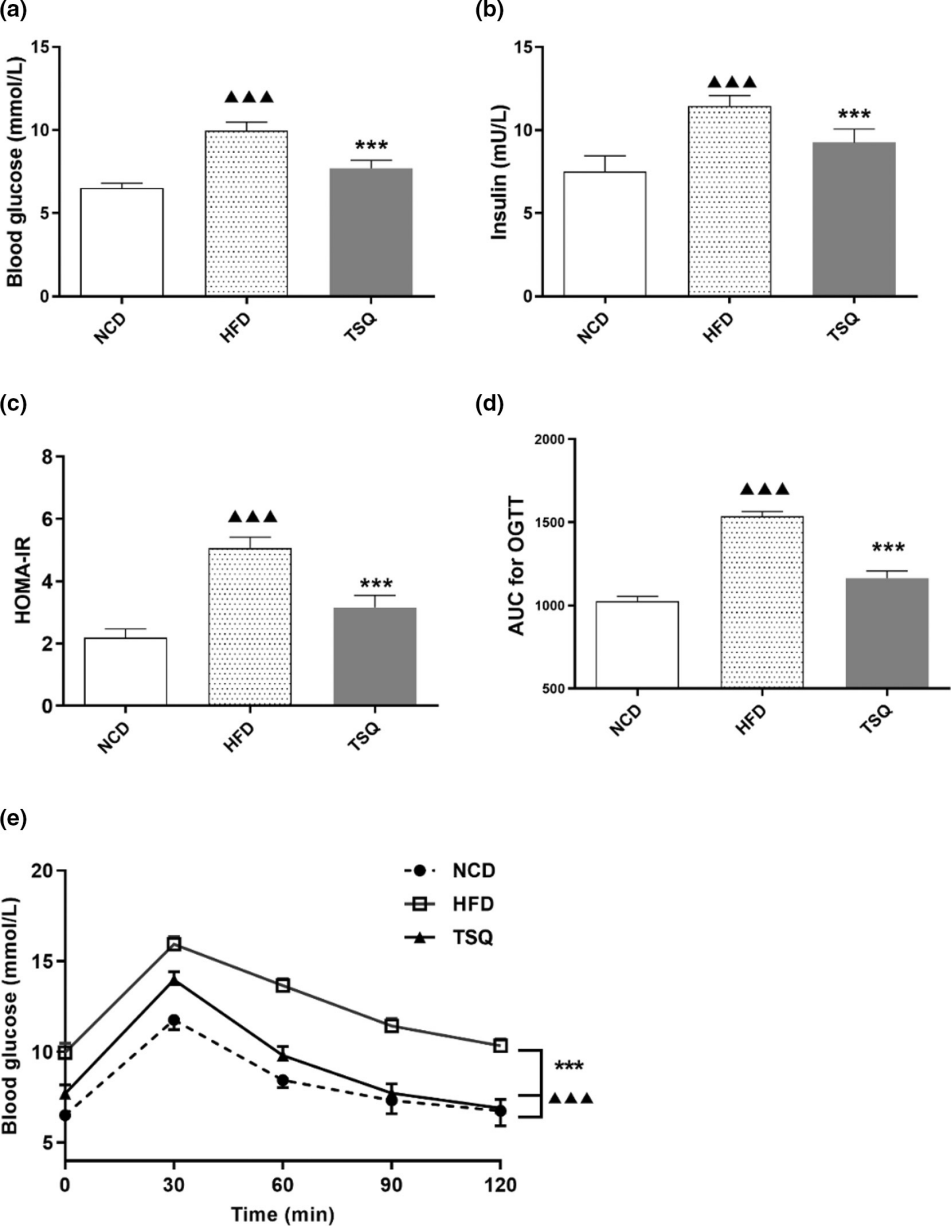
Preventive effects of TSQ on the development of insulin resistance in high‐fat diet‐fed rats. (a) Fasting blood glucose, (b) fasting insulin, (c) homeostasis model assessment for insulin resistance (HOMA‐IR), (d) area under curve (AUC) of OGTT, and (e) oral glucose tolerance test (OGTT) of rats in each group. ^▲▲▲^ *p* < .001, ^▲▲^ *p* < .01, ^▲^ *p* < .05 statistically significant differences compared with NCD group; ***p < .001, **p < .01, *p < .05, statistically significant differences compared with HFD group

High‐fat diet feeding lead to an increase in fasting serum glucose level, insulin level, and HOMA‐IR index, which suggested insulin resistance in HFD‐fed rats. However, TSQ administration significantly reduced the serum glucose and insulin levels along with a decrease in the HOMA‐IR index (*p* < .001) (Figure 2a–c). Moreover, 7 weeks of TSQ intervention could effectively improve glucose tolerance, as demonstrated by reduced blood glucose levels in OGTT (*p* < .001) (Figure 2d,e). Consistent with these results, the glucose total AUC of the TSQ group was extremely decreased compared with that of the HFD group (*p* < .001) (Figure 2e). Together, these results indicated that TSQ supplementation improved glucose tolerance and insulin resistance in diet‐induced obese rats.

### 3.5 TSQ attenuates metabolic endotoxemia, systemic inflammation, and serum lipid profile

**FIGURE 3:**
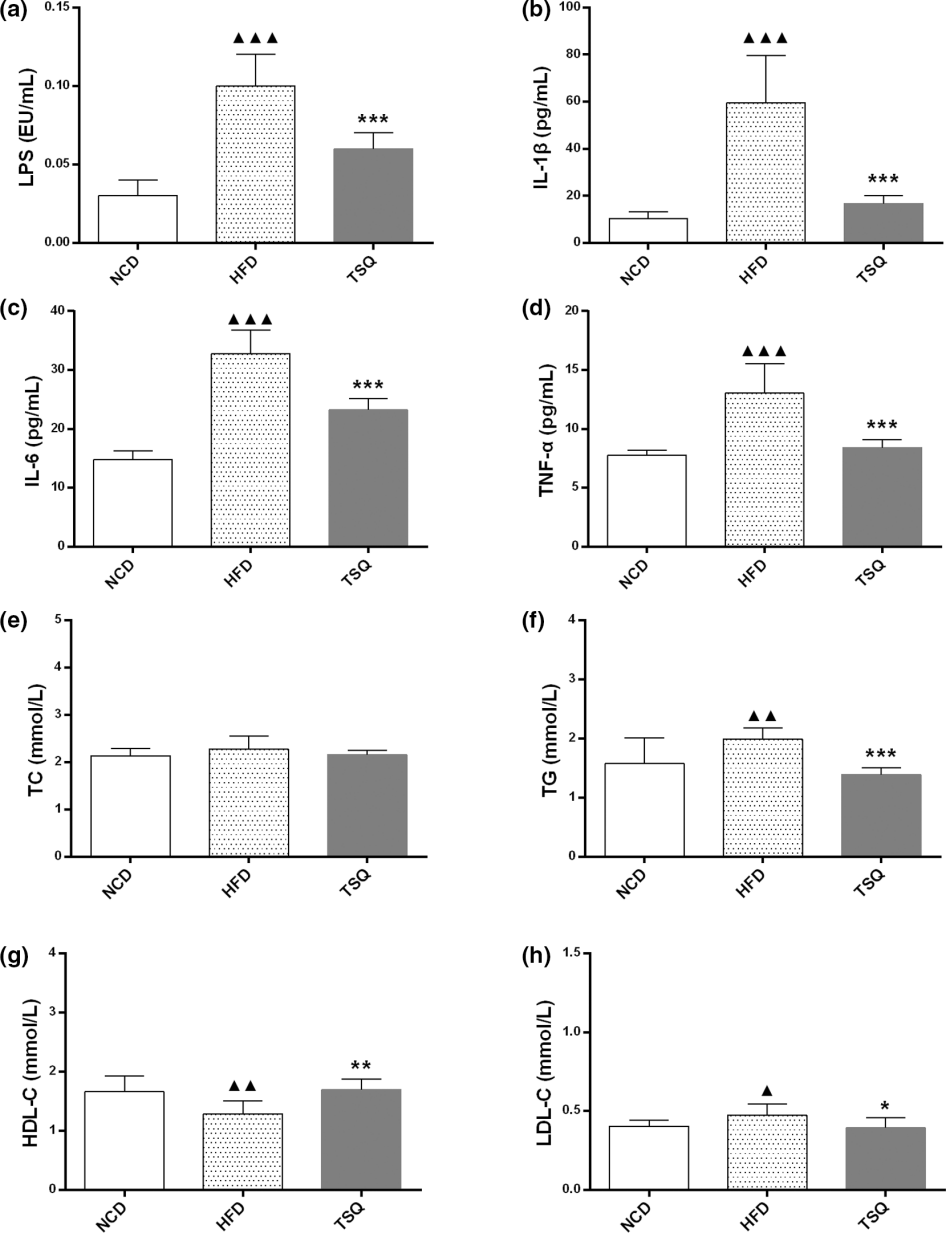
TSQ regulated metabolic endotoxemia, systemic inflammation, and serum lipid profile in rats with high‐fat induced obesity. (a) LPS, (b) IL‐1β, (c) IL‐6, (d) TNF‐α, (e) TC), (f) TG, (g) HDL‐C, and (h) LDL‐C levels from serum of rats in each group. ^▲▲▲^ *p* < .001, ^▲▲^ *p* < .01, ^▲^ *p* < .05 statistically significant differences compared with NCD group; ***p < .001, **p < .01, *p < .05, statistically significant differences compared with HFD group

The levels of metabolic endotoxemia, proinflammatory cytokines, and the lipid profile in serum significantly increased after HFD feeding. The administration of HFD resulted in dramatically increased lipopolysaccharide (LPS) levels in serum compared with the normal diet (0.10 ± 0.02 vs. 0.03 ± 0.01 EU/ml, *p* < .001), while supplementation of TSQ significantly decreased HFD‐induced LPS appearance in the serum (0.06 ± 0.01 vs. 0.10 ± 0.02 EU/ml, *p* < .001) (Figure 3a). The serum proinflammatory cytokines level in the HFD group were also significantly higher than NCD group. Compared with those of the NCD group, the HFD increased serum IL‐1β, IL‐6, and TNF‐α levels by approximately 4.8, 1.2, and 1.7 times, respectively. (*p* < .001). Administration of TSQ significantly decreased serum proinflammation markers levels, especially the level of IL‐1β, which was down to the level observed in the NCD group (*p* < .001) (Figure 3b–d). In addition, animals of the HFD group exhibited dyslipidemia compared with NCD group. The contents of LDL‐C, TG, and TC in the HFD group were significantly higher than in the NCD group (*p* < .05), while HDL‐C levels in the HFD group were significantly lower than in the NCD group (*p* < .05) (Figure 3e–h). TSQ administration effectively corrected the abnormalities of blood lipid parameters and significantly decreased TG, TC, and LDL‐C concentrations (Figure 3e–h). Overall, our results suggested that HFD led to metabolic endotoxemia, promoted systemic inflammation, and fat accumulation, whereas TSQ successfully corrected these disorders.

### 3.6 TSQ modulates the structure of the gut microbiota

**FIGURE 4:**
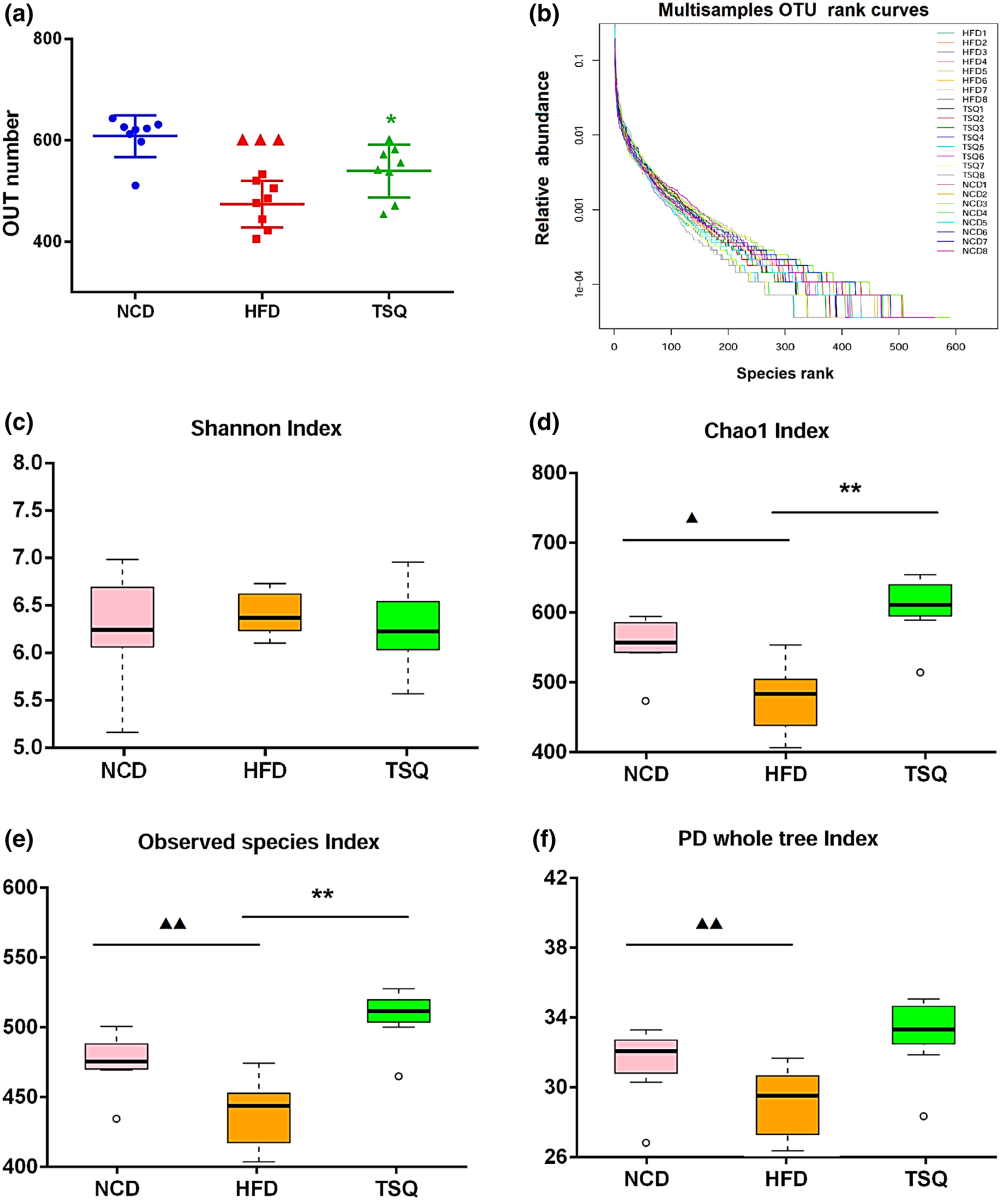
Effects of TSQ on the diversity and enrichment of gut microbiota in high‐fat induced obesity rats. (a) Operational taxonomic units (OTU) number of gut microbiota in three groups and (b) OTU rank curves of gut microbiota for each sample. (c–f) The Shannon index, Chao1 index, observed species index, and PD whole tree index of each group. ^▲▲▲^ *p* < .001, ^▲▲^ *p* < .01, ^▲^ *p* < .05 statistically significant differences compared with NCD group; **p < .01, *p < .05, statistically significant differences compared with HFD group

To further investigate the regulation effect of TSQ on gut microbial composition, the V_3_–V_4_ hypervariable region of the 16S rDNA gene was sequenced, resulting in 921,008 raw reads from 24 samples. After denoising and filtering, 864,927 clean tags were used for subsequent analysis. Based on a 97% similarity level, all of the effective reads were clustered into OTU. As shown in Figure 4a, compared with that of the NCD group, the HFD significantly reduced the number of OTUs (*p* < .001). Furthermore, other microbial diversity indices, such as the Shannon index, Chao1 index, observed species index, and PD whole tree index were increased by TSQ administration (Figure 4c–f).

**FIGURE 5:**
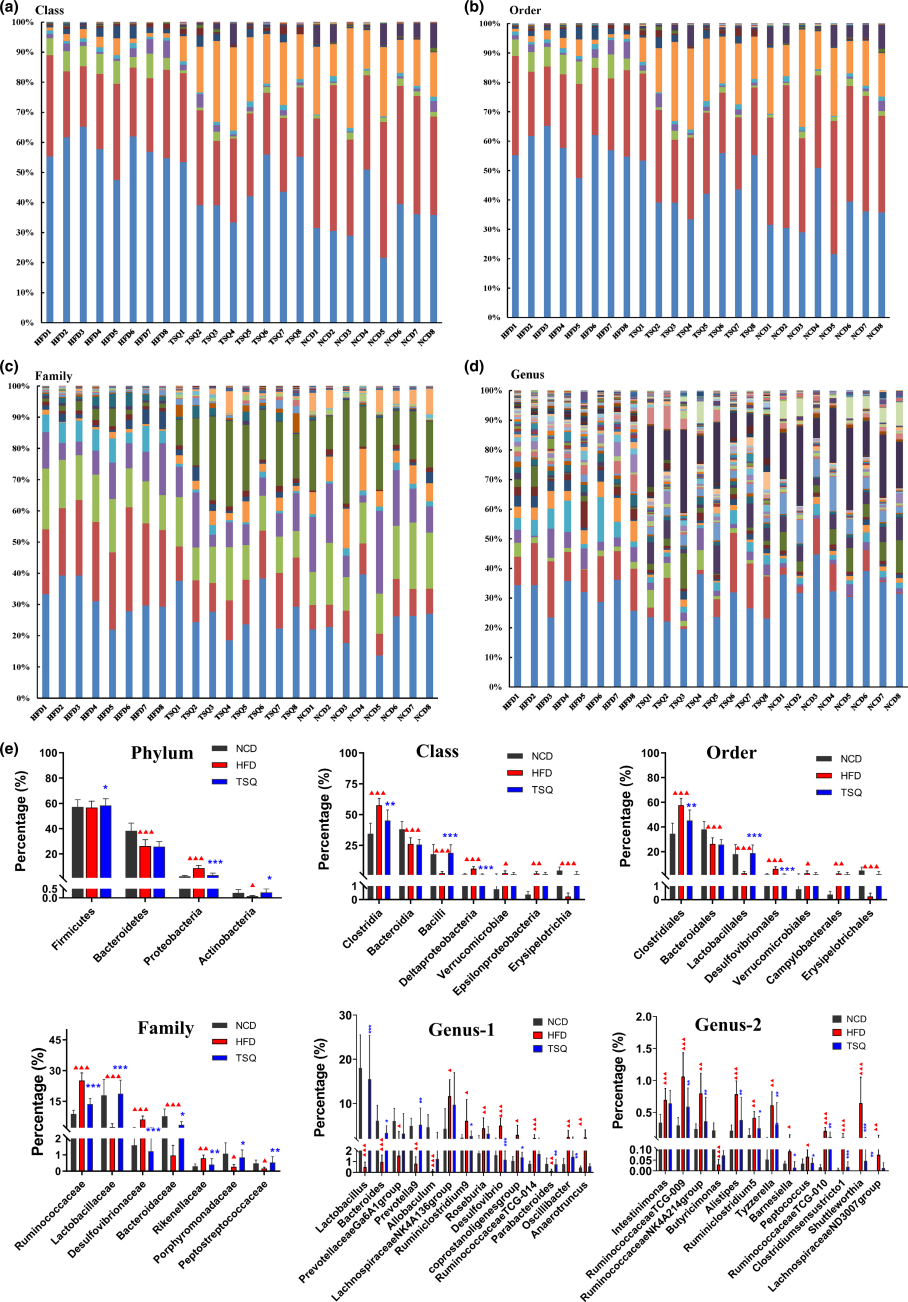
Effects of TSQ on the composition of gut microbiota at different levels in high‐fat induced obesity rats. (a) Class, (b) order, (c) family, (d) genus, and (e) representative flora with significant differences at different levels in each group. ^▲▲▲^ *p* < .001, ^▲▲^ *p* < .01, ^▲^ *p* < .05 statistically significant differences compared with NCD group; ***p < .001, **p < .01, *p < .05 statistically significant differences compared with HFD group

We further evaluated the overall regulation effects of HFD and TSQ on the gut microbiota composition. At the phylum level, the gut microbial communities of rats were dominated by bacteria from Firmicutes, Bacteroidetes, and Proteobacteria (Figure 5e). Roopchand et al. (2015) found an increased ratio of Firmicutes versus Bacteroidetes in the HFD group. Our results showed that HFD feeding significantly decreased the abundance of Bacteroidetes. However, TSQ restored the difference to similar levels as in the NCD group. Moreover, we detected a significant increase in the count of Proteobacteria in the HFD group and a decrease in the TSQ group. Consistent with the result of the phylum level, the abundance of Deltaproteobacteria and Epsilonproteobacteria were also evaluated by HFD at the class level. Furthermore, class‐level results showed a significantly higher level of Clostridia and Verrucomicrobiae as well as significantly lower levels of Bacilli, Bacteroidia, and Erysipelotrichia in HFD group. The percentage levels of Clostridia and Bacilli were reverted to normal in the TSQ group (Figure 5b, f_2_). At the order level, HFD significantly lowered the levels of Bacteroidales, Lactobacillales, and Erysipelotrichales, and increased the levels of Clostridiales, Desulfovibrionales, Verrucomicrobiales, and Campylobacterales than NCD. After TSQ supplementation, the levels of Lactobacillales, Clostridiales, and Desulfovibrionales in the TSQ group were close to NCD group. At the family level, TSQ treatment restored bacterium to near normal levels such as Lactobacillaceae, Bacteroidaceae, Porphyromonadaceae, Peptostreptococcaceae, Ruminococcaceae, Desulfovibrionaceae, and Rikenellaceae. At the genus level, we observed significantly lower levels of 7 genera and higher levels of 20 genera in the HFD group than in the NCD group. Again, TSQ supplements prevented these changes induced by HFD.

**FIGURE 6:**
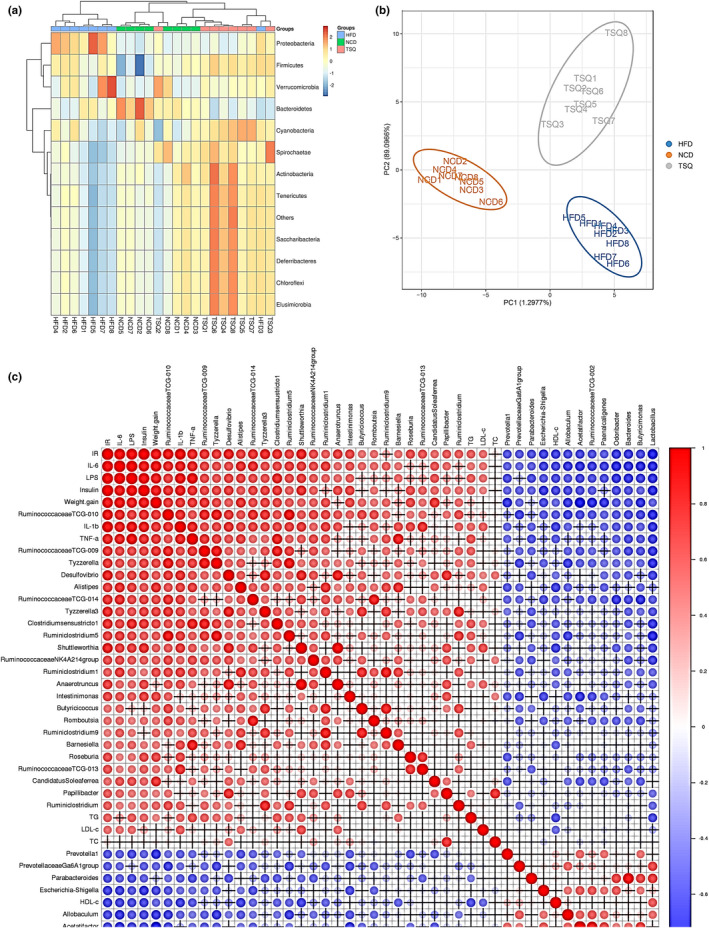
Overall structure of gut microbiota in response to HFD and TSQ administration. (a) Clustering analysis and (b) PLS‐DA analysis in each group. (c) Correlations between key gut microbiota and serum biochemical measurements by Pearson's correlation analysis. The correlation is represented by the depth of the color and the size of the circle. Red indicates positive correlation and blue indicates negative correlation

The intestinal microbiota structural variation were analyzed using multivariate statistical methods to clarify the overall regulatory role of TSQ on HFD‐induced obesity rats. Clustering analysis (Figure 6a) and PLS‐DA (Figure 6b) explained the similarities and variations among the NCD, HFD, and TSQ groups, among which the first two components explained 89.10% of the total variance in PLS‐DA. The clustering heatmap of alterations in the intestinal microbiota suggested that the TSQ and NCD groups were clustered to a class, while the HFD group was near, showing that TSQ intervention can normalize the flora at the phylum level (Figure 6a).

### 3.7 Correlations between key gut microbiota and body weight gain, serum endotoxemia, inflammation, and lipid profile

Pearson's correlation analysis was applied to analyze the associations between the overall composition of intestinal flora and obesity‐related metabolic disorders. By the correlation analysis, we identified 35 key genera, which were significantly correlated with features of obesity (body weight gain), dyslipidemia (TC, TG, HDL‐C, and LDL‐C), proinflammatory cytokines (serum IL‐6, IL‐1β, and TNF‐α), and LPS (Figure 6c). Among them, 23 key genera were positively correlated with obesity‐related properties. In contrast, the others were negatively correlated with the features of obesity. Importantly, our study identified potential links between variation in microbiota composition and associations with insulin resistance, IL‐6, and LPS levels in serum. However, the exact relationship needs to be explored in future studies (e.g., fecal microbiota transplantation experiment). Accordingly, we can conclude that the modulation effect of gut microbiota components by TSQ is correlated with improving metabolic disorders, especially for inflammation levels and insulin resistance in obese rats.

## 4 DISCUSSION

Obesity is regarded as a major public health problem accompanying a series of chronic diseases, and is also thought to be the risk factor of various metabolic diseases (Blüher, 2019). The prevalence of obesity is related to many factors, among which metabolic factors, HFD, and unhealthy lifestyles are considered to be important causes of obesity. Reducing calorie diet intake and increasing physical activity are currently important strategies for the treatment and control of weight gain (Valenti et al., 2020). A pharmacologic treatment is recommended when behavioral approach is not sufficient to achieve the goal of weight control. Currently, many side effects of recommended drugs were reported on obesity treatment such as diarrhea, stool incontinence, flatulence, bloating, and indigestion (Kang & Park, 2012). Therefore, bioactive compounds derived from food or natural products have attracted great attention, and the research on natural substance has provided new strategies for preventing obesity. A growing body of experimental evidence has proved that secondary metabolites of quinoa possess a wide range of biological activities (Lin et al., 2019). Among these, the antioxidant and immune‐regulatory activities have attracted the increasing attention. However, in this study, we evaluated the potential function of TSQ in improving obesity‐related inflammation and insulin resistance, as well as regulating intestinal microflora dysbiosis.

Aqueous two‐phase extraction system is widely used in separation and purification of small molecules due to demixing effect, extraction selectivity, the environmental‐friendly features (Zhao et al., 2020). In this study, we adopted this approach and applied it to obtain high yield of total saponins. Compared with traditional extraction methods, ATPS is a more effective process to extract total saponins from the quinoa brans. Further assessment of the safety of TSQ showed that no side or adverse effects were displayed during the trial. In addition, there was no erythrocyte aggregation when the concentration of TSQ was <1.0 mg/ml. In summary, our research initially showed that it was suitable for oral administration in rats at the concentration <1.0 mg/ml. However, further clinical studies are warranted to investigate safety and efficacy upon systemic administration (i.v.).

HFD induced an elevation of weight in the body and organs as well as abnormal serum lipid level. In the current experiment, we investigated the effect of oral administration with 100 mg/kg TSQ for 7 weeks on the development of obesity. Compared with HFD group, TSQ intervention significantly reduced body weight gain and epididymal and retroperitoneal WAT mass, which strongly revealed the health‐promoting effect of TSQ on diet‐induced obesity. A previous study reported that WAT was the primary location for energy storage for the body under the conditions of excess energy (Flier, 1995). Moreover, the abnormal lipid metabolism was observed in HFD rats, which was characterized by uncontrolled serum levels of TC, TG, HDL‐C, and LDL‐C. Our results showed that TSQ supplementation effectively corrected the abnormalities of blood lipid parameters and result in a significant decrease in serum concentrations of TG, TC, and LDL‐C. In addition, we have observed that saponin extracts showed satisfying effect on the improvement of energy metabolism in HFD‐fed rats. Long‐term TSQ supplementation obviously reduced the fasting blood glucose and insulin as well as HOMA‐IR index compared to HFD group. Taken together, our observations indicated that TSQ exhibited anti‐obesity effects, evidenced by TSQ supplementation significantly reduces adipose accumulation and improves energy metabolism in obese rats.

Accumulated evidence has causally linked obesity to a chronic low‐grade inflammatory state, which may be mediated by the alteration of intestinal permeability (Fukui, 2016). Thus, the reducing LPS, one of the key contributing factors that affect gut permeability and systemic inflammation, acts as a pivotal strategy for the prevention and treatment of obesity‐related chronic metabolic diseases. The present study showed that TSQ administration significantly decreased proinflammatory cytokines TNF‐α, IL‐1β, and IL‐6 levels, resulting in the anti‐inflammatory effect of TSQ. Combined with other related reports, we speculated that TSQ exerted the anti‐obesity effect by reducing systemic inflammation (Xie et al., 2018). Saponins are naturally occurring surface‐active glycosides, whose structure consist of a triterpenoid or steroid non‐polar aglycone linked to one or more oligosaccharide moieties through a glycosidic linkage (El Aziz et al., 2019). The representative characteristic of saponins includes low bioavailability, difficult absorption, and long retention times in the intestine. Such features determine they are prone to be metabolized and hydrolyzed by intestinal microorganisms, and performing the functions of secondary glycosides or aglycones (Navarro del Hierro et al., 2019). Previous research found that the seeds of the pseudo‐cereal quinoa were rich in triterpenoid saponins (Medina‐Meza et al., 2016). Our previous study indicated that quinoa saponin fractions mediated these anti‐inflammatory actions by inhibiting the release of proinflammatory cytokines in vitro (Yao, Yang, et al., 2014). Therefore, it is speculated that regulating the gut microbiota can be one of the mechanisms of TSQ's anti‐obesity effect, because the oral absorption is limited by intestine.

A bidirectional regulation effect of TSQ on gut microbes was found in our research. Above all, TSQ has the capacity to selectively promote the growth of some beneficial bacteria. Bacilli has been reported to impart a positive impact on the body based on its regulatory and health‐promoting actions that produce bioactive secondary metabolites including low‐molecular‐weight regulatory agents, proteins, and peptides (Ilinskaya et al., 2017). We found that TSQ administration strongly inhibited the decrease of the relative abundance of Bacilli caused by HFD. Therefore, TSQ may play a role in intestinal health promotion by inhibiting the reduction of the probiotic activity of bacteria. Recently, researchers have also found that weight loss was strongly associated with an increased proportion of *Lactobacillus* (Kim et al., 2017). Our results suggested that TSQ treatment markedly increased the relative abundance of *Lactobacillus*, suggesting a positive link between body weight management and *Lactobacillus*. Another interesting result was the beneficial effect of TSQ on butyrate‐producing bacterium, such as *Allobaculum* and *Butyricimonas*. Butyrate is a microbial metabolite of dietary fiber with broad health‐protecting effects, including anti‐inflammatory, enhancing intestinal barrier function and regulating numerous biological processes serve as a signaling molecule (Wu et al., 2012). Butyrate was also reported to reduce gut mucosal permeability, increase transepithelial electrical resistance, and impede PEG translocation in the heat‐damaged rat colon (Conterno et al., 2011). Collectively, the current findings strongly suggest that TSQ may contribute health‐promoting effects through an enhancement of the relative abundance of anti‐inflammation‐related gut microbiota.

The results of our study demonstrated that TSQ exerted an anti‐obesity effect by decreasing the production of LPS via inhibiting the overgrowth of harmful bacteria. LPS, known as endotoxins, are mainly found in the outer membrane of gram‐negative bacteria. Growing evidence suggests that the leakage of intestinal LPS evokes chronic inflammation and obesity (Zhang et al., 2019). In this study, we found that the relative abundance of LPS‐producing bacteria, *Desulfovibrio*, was decreased in TSQ group. It was consistent with a previous study that the prebiotic intervention could reduce the abundance of Desulfovibrionaceae in long‐term HFD feeding individuals (Zhai et al., 2018). In addition, we detected a significant decrease in the count of Proteobacteria in the TSQ group. Proteobacteria has been suggested as a marker of unstable microbial communities and potential proinflammation (Rizzatti et al., 2017). Studies suggest that there is a considerable link between elevated Proteobacteria levels and metabolic disorders, such as obesity and T2D (Suez et al., 2014). An instant high abundance of Proteobacteria and low‐grade inflammation are distinct feature of intestinal microbes in Toll‐like receptor 5‐deficient mice (Carvalho et al., 2012). Our research showed that TSQ might exert anti‐inflammatory activity by inhibiting the prevalence of inflammatory Proteobacteria.

## 5 CONCLUSIONS

In summary, our findings suggested that TSQ treatment attenuated HFD‐induced obesity and related low‐grade chronic inflammation in rats. TSQ exhibited promising effects on suppressing body weight gain and visceral white adipose accumulation, improving glucose intolerance and insulin resistance, and attenuating metabolic endotoxemia, systemic inflammation, and the serum lipid profile. Furthermore, TSQ administration resulted in a striking growth of beneficial bacteria while inhibiting the proportion of harmful bacteria. These new findings indicated that the gut microbiota might be involved in the anti‐obesity effects of TSQ. Taken together, our results implied that TSQ might be used as a bioactive ingredient with prebiotic properties to prevent obesity and associated disorders.

## CONFLICT OF INTEREST

The authors have declared no potential conflicts of interest.

## ETHICAL APPROVAL

Animal experiments were approved by the Animal Experimentation Ethics Committee of the Chengdu University, complied with the ARRIVE guidelines, and carried out in accordance with the EU Directive 2010/63/EU for animal experiments.

## Data Availability

Data openly available in a public repository that issues datasets with DOIs
